# Risk factors affecting post-pubertal high serum follicle-stimulating hormone in patients with hypospadias

**DOI:** 10.1007/s00345-019-02687-w

**Published:** 2019-02-28

**Authors:** Kimihiko Moriya, Michiko Nakamura, Masafumi Kon, Yoko Nishimura, Yukiko Kanno, Takeya Kitta, Nobuo Shinohara

**Affiliations:** Department of Renal and Genitourinary Surgery, Hokkaido University Graduate School of Medicine, North-15, West-7, Kita-Ku, Sapporo, 060-8638 Japan

**Keywords:** Hypospadias, Undescended testis, Follicle-stimulating hormone, Spermatogenesis

## Abstract

**Purpose:**

The factors affecting spermatogenesis in adulthood in patients with hypospadias (HS) are not clearly understood. In the present study, risk factors affecting post-pubertal high serum follicle-stimulating hormone (FSH) were evaluated in patients with HS.

**Materials and methods:**

Among those with a history of HS surgery, patients in whom endocrinological evaluation regarding pituitary–gonadal axis was performed at 15 years of age or older between March 2004 and April 2018 were enrolled in the present study. High serum FSH was defined as greater than 10 mIU/ml. The severity of HS was divided into mild and severe. Factors affecting the post-pubertal high serum FSH were estimated.

**Results:**

Seventy-nine patients were included in the present study. The severity of HS was mild in 35 and severe in 44. History of undescended testis (UDT) was confirmed in 12. High serum FSH was detected in nine. On logistic regression model analysis, a history of UDT was the only significant factor for high serum FSH. The incidence of high serum FSH in patients with UDT was significantly higher than that in those without UDT (58.3% vs 7.5%, *p* < 0.01). When stratified by severity of HS and the presence of UDT, high serum FSH was detected in 70% in patients with severe HS and UDT, whereas less than 10% in other groups.

**Conclusions:**

A history of UDT was a significant factor for post-pubertal high serum FSH in patients with HS. Accordingly, the presence of UDT may be a marker for impaired spermatogenesis in patients with HS, especially in severe cases.

## Introduction

Hypospadias (HS) is one of the most common congenital anomalies in male children, occurring in 0.52–8.2 of every 1000 live births [1, 2]. Although there has been great surgical interest in short-term outcomes, such as fistula or stricture, information about the long-term outcomes of this anomaly is limited.

A multifactorial etiology, including genetic, endocrine, and environmental factors, is considered to be involved in the genesis of this anomaly [3]. As male urethral development is androgen-dependent, there may be a risk of gonadal and reproductive disorders in patients with HS at puberty or later. Our previous study demonstrated endocrinological abnormalities in the pituitary–gonadal axis in patients with mild and severe HS [4]. In other previous reports, severe HS or associated genital abnormality, such as micropenis, undescended testis (UDT), or testicular tumor, were found to be risk factors for testicular and reproductive dysfunction [5–7]. In addition, birth weight (BW) or body mass index (BMI) has also been reported to affect the semen quality in the general population [8–13]. However, factors affecting spermatogenesis in patients with a history of HS in adulthood are not clearly understood.

In the present study, we investigated the risk factors affecting reproductive function in patients with HS. Given the difficulty in obtaining semen samples from asymptomatic patients, serum follicle-stimulating hormone (FSH) was used as a surrogate marker of spermatogenic function because previous studies demonstrated that FSH level in adults seems to be correlated with spermatogenetic activity [14, 15] and to be a predictive marker for the sperm retrieval rate in patients with non-obstructive azoospermia [16, 17], although exact role of FSH remains unclear.

## Patients and methods

All patients with a history of HS surgery at our institute or affiliated hospitals were recommended to be followed for regular visits until the post-pubertal period. Medical charts of patients who visited our clinic for regular follow-up after HS surgery were retrospectively reviewed. Among them, patients who were born after July 1986 and in whom endocrinological evaluation regarding the pituitary–gonadal axis, including luteinizing hormone, FSH, and testosterone, was performed at 15 years of age or older between March 2004 and April 2018 were enrolled in the present study. If multiple endocrinological evaluations were carried out in one patient at 15 years of age or older, the final evaluation was used in the present study. Patients with obvious disorders of sex development or chromosomal abnormalities were excluded. High serum FSH was defined as greater than 10 mIU/ml. Severity of HS was divided into mild and severe based on the necessity of transecting urethral plate for correction of chordee deformity according to Koyanagi et al. [18]. Factors affecting post-pubertal high serum FSH were estimated.

JMP^®^pro version 13 was used for all statistical analyses. Statistical analysis was performed using Fisher’s exact probability test or logistic regression model analysis for the determination of risk factors. *p* < 0.05 was considered as significant.

The present study was approved by the Institutional Review Board of Hokkaido University Hospital (Approval number: 017–0066).

## Results

### Patient characteristics (Table 1)

Seventy-nine patients were included in the present study. Since endocrinological evaluation regarding the pituitary–gonadal axis was routinely performed at puberty or later, all patients with HS who visited during study period at 15 years of age or older were enrolled. The median BW, age at endocrinological evaluation, BMI, and Tanner pubic hair stage at evaluation were 2410 g (interquartile range 1626–3034), 17.5 years (range 15.0–27.0), 21.3 kg/m^2^, and stage 5 (3–5), respectively. The severity of HS was mild in 35 and severe in 44. History of UDT was confirmed in 12 (unilateral in 5 and bilateral in 7/congenital in 3 and ascending in 9). The incidence of UDT was 2 of 35 (5.7%) with mild HS and 10 of 44 (22.7%) with severe HS (*p* = 0.06).

**Table 1:** Patient characteristics

Birth weight (g) IQR/median ± SD	1626–3034 2410 ± 877 (unknown 4 pts)
Severity of HS	Mild: 35 pts Severe: 44 pts
Undescended testis	12 pts
Unilateral	5 pts
Bilateral	7 pts
Congenital	3 pts
Ascending	9 pts
Age at evaluation (years) (median ± SD)	15.0–27.0 (17.5 ± 2.1)
Tanner stage at evaluation	III: 2 pts, IV: 11 pts, V: 60 pts (unknown: 6 pts)
Body Mass Index at evaluation (kg/m^2^) (median ± SD)	12.2–32.6 (21.3 ± 3.7)
Serum FSH (mIU/ml) (median ± SD)	1.3–112.4 (4.7 ± 18.2)
< 10 mIU/ml	67 pts
10-mIU/ml	12 pts

*IQR* Interquartile range

### Incidence and risk factors of high serum FSH

High serum FSH was detected in 12 patients. On logistic regression model analysis, a history of UDT was the only significant factor for high serum FSH (Table 2). The incidence of high serum FSH in patients with UDT was significantly higher than that in those without UDT (58.3% vs 7.5%, *p* = 0.0002) (Table 3). When compared between unilateral and bilateral UDT, or between congenital UDT and ascended testis, the incidence of high serum FSH was 3/5 (60.0%) versus 4/7 (57.1%), and 1/3 (33.3%) versus 6/9 (66.7%), respectively. However, these differences were not statistically significant.

**Table 2:** Risk factors for high serum FSH

	Odds ratio (95% confidence interval)	*p*
Birth weight	0.612 (0.0429–7.017)	n.s
Severity of HS		
Mild	Reference	
Severe	2.743 (0.744–13.180)	n.s
Undescended testis		
No	Reference	
Yes	17.36 (4.196–82.242)	< 0.0001
Age at evaluation	1.043 (0.028–38.681)	n.s
Tanner stage at evaluation		
V	Reference	
V >	5.250 (0.7916–13.343)	n.s
Body Mass Index at evaluation	3.055 (0.0560–166.738)	n.s

**Table 3:** Impact of undescended testis on high serum FSH

Undescended testis	Incidence of high serum FSH	*p*
Yes	7/12 (58.3%)	0.0002
No	5/62 (7.5%)	

### Impact of severity of HS

**Fig. 1:**
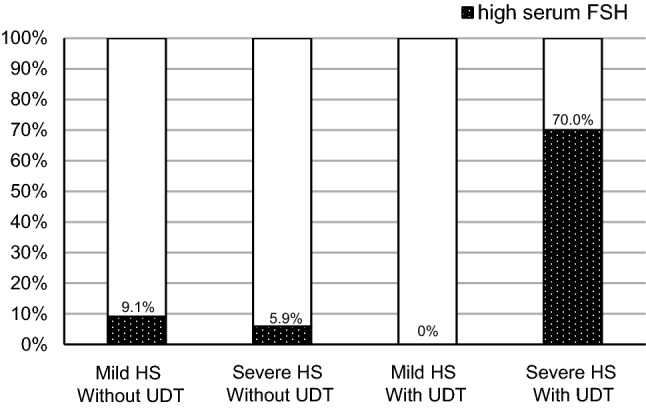
Incidence of high serum FSH stratified by severity of HS and presence/absence of UDT. While the incidence of high serum FSH was as low as 10% in patients with mild HS without UDT (*n* = 33), in patients with severe HS without UDT (*n* = 34) or in patients with mild HS and UDT (*n* = 2), 70% showed high serum FSH in patients with severe HS and UDT (*n* = 10)

When patients were divided by severity of HS and the presence/absence of UDT, median value of serum FSH was 3.8 mIU/ml in patients with mild HS without UDT (*n* = 33), 5.4 in patients with severe HS without UDT (*n* = 34), 2.4 in patients with mild HS and UDT (*n* = 2), or 12.4 in patients with severe HS and UDT (*n* = 10), respectively. The rate of high serum FSH was 9.1% in patients with mild HS without UDT, 5.9% in patients with severe HS without UDT, none in patients with mild HS and UDT, or 70% in patients with severe HS and UDT, respectively (Fig. 1).

## Discussion

The present study demonstrated that concomitant UDT was the only risk factor for high serum FSH in patients with HS in the post-pubertal period. The presence of UDT would be a marker for impaired spermatogenesis in patients with HS, especially in severe cases.

Previous reports on spermatogenesis in patients with HS in the post-pubertal period are limited. Asklund et al. [6]. found that the semen quality in men with HS and additional genital disorders (HAGD) was significantly poorer than that in those with isolated HS. The FSH level was also higher in men with HAGD than in those with isolated HS. Rey et al. [5]. reported that the risk of impaired Sertoli and Leydig cell function was higher in patients with HS and associated genital malformation than in those with isolated HS. Accordingly, as previously reported, some patients with HS, especially with additional genital disorders, are classified as having testicular dysgenesis syndrome [19]. These findings are consistent with the present study though patients’ characteristics such as age at evaluation or severity of HS were different from previous studies.

Regarding the severity of HS, the present study demonstrated that it was not a significant factor affecting serum FSH. Kumar et al. [7]. recently reported poorer semen quality in patients with severe HS. However, much attention was not paid to associated genital anomalies such as UDT in their study. The current study and our previous report revealed that the incidence of high serum FSH was as low as 10%, and was similar in isolated mild or severe HS [4]. And patients with severe HS tended to have UDT more frequently than those with mild HS in the present study. Accordingly, difference in the rate of concomitant UDT, not the severity of HS, was considered to affect the outcome of the study by Kumar et al. though the possibility that patients with both severe HS and UDT were at a higher risk of worse semen quality cannot be denied. Although gonadal dysfunction has been reported in both mild and severe HS [4, 20, 21], there are few reports on the differences in reproductive function based on the severity of HS. Thus, the impact of HS severity on reproductive function still remains unclear. Further studies are warranted.

In the current study, a history of UDT was the only significant risk factor for high serum FSH in patients with HS. As UDT itself carries the risk of impaired spermatogenesis, it is unknown whether concomitant HS increases the risk of impaired spermatogenesis. Previous studies demonstrated that the incidence of high serum FSH in patients with isolated bilateral UDT was significantly higher than that in patients with isolated unilateral UDT [22–24]. On the other hand, the incidence of high serum FSH in our study was similar in patients with HS and unilateral or bilateral UDT, although the number of patients was limited. As no difference was noted between patients with isolated unilateral UDT and the normal population regarding fertility [25], unilateral UDT in patients with HS may differ in pathology from isolated unilateral UDT in terms of spermatogenesis. Longer follow-up with lager number of patients are necessary to clarify this issue.

Nakamura et al. reported that a risk factor for testicular microlithiasis in patients with HS was concomitant UDT [26], which was also found to be a risk factor for high serum FSH in the current study. They found that the incidence of TM in patients with and without UDT was 43.8% and 9.5%, respectively, which was similar with the incidence of high serum FSH in those with and without UDT (58.3% or 7.5%, respectively) in the present study. In addition, Asklund et al. reported that men with HS and testicular microlithiasis had a lower sperm concentration compared with men without microlithiasis [6]. As more than 40% of patients with HS and UDT did not have high serum FSH, testicular ultrasound may be beneficial to determine the risk of high serum FSH or impaired spermatogenesis in patients with HS and UDT. The prevalence of testicular microlithiasis in patients with UDT was reported to increase with time [27]; therefore, further studies are necessary to clarify the effects and optimal timing of testicular ultrasonography in patients with HS and UDT.

Low BW is a well-known risk factor for HS [28–30]. In previous reports, low BW was also found to be a risk factor for low semen quality in the general population, albeit controversial [8–11]. The current study demonstrated that BW was not associated with serum FSH. As the median BW for our patients was 2410 g, indicating that more than half of the included patients had a low birth weight, it may be difficult to reach significance with this cohort. BMI was also reported to be a risk factor for poor semen quality, although there has been some debate [11–13]. However, BMI was not associated with serum FSH in our study. This may be due to the relatively low BMI at evaluation (median 21.3 kg/m^2^) in our cohort.

There are several limitations in the present study. First, semen analysis was not performed because of the difficulty in obtaining semen samples form asymptomatic patients. Although previous studies demonstrated that FSH level in adults seems to be correlated with spermatogenetic activity and to be a predictive marker for the sperm retrieval rate in adults with non-obstructive azoospermia, FSH alone was reported to be insufficient for prediction [17]. In addition, median age of patients included in the current study was 17.5, slightly younger to discuss the relation between serum FSH and semen quality based on previous reports. Second, there was no control in this study. Accordingly, although risk factors for high serum FSH among patients with HS were analyzed in the current study, it was not possible to demonstrate their impact of HS regarding spermatogenesis. A previous study reported that the semen quality of the majority of men with isolated HS was within the control range, but a slightly abnormal hormonal status was noted [6]. Thus, the outcome using semen analysis may differ from that of the current study. Last, the number of patients included in the present study was relatively low, especially for patients with UDT.

## Conclusion

Our study demonstrated that concomitant UDT was the only risk factor for high serum FSH in the post-pubertal period in patients with HS. Accordingly, the presence of UDT may be a marker for impaired spermatogenesis in patients with a history of HS especially in severe cases. This information is useful for physicians when following up patients with HS after surgery.
